# Six Decades of Research on Human Fetal Gonadal Steroids

**DOI:** 10.3390/ijms22136681

**Published:** 2021-06-22

**Authors:** Stéphane Connan-Perrot, Thibaut Léger, Pauline Lelandais, Christèle Desdoits-Lethimonier, Arthur David, Paul A. Fowler, Séverine Mazaud-Guittot

**Affiliations:** 1 Univ Rennes, Inserm, EHESP, Irset (Institut de Recherche en Santé, Environnement et Travail), UMR_S 1085, 35000 Rennes, France; stephane.connan-perrot@etudiant.univ-rennes1.fr (S.C.-P.); pauline.lelandais@univ-rennes1.fr (P.L.); christele.desdoits-lethimonier@univ-rennes1.fr (C.D.-L.); Arthur.DAVID@ehesp.fr (A.D.); 2 Fougères Laboratory, French Agency for Food, Environmental and Occupational Health & Safety (ANSES), CEDEX, 35306 Fougères, France; thibaut.leger@anses.fr; 3 Institute of Medical Sciences, University of Aberdeen, Foresterhill, Aberdeen AB25 2ZD, UK; p.a.fowler@abdn.ac.uk

**Keywords:** detection, quantification, testis, ovary, steroidogenesis, androgens, estrogens, human, fetal

## Abstract

Human fetal gonads acquire endocrine steroidogenic capabilities early during their differentiation. Genetic studies show that this endocrine function plays a central role in the sexually dimorphic development of the external genitalia during fetal development. When this endocrine function is dysregulated, congenital malformations and pathologies are the result. In this review, we explain how the current knowledge of steroidogenesis in human fetal gonads has benefited from both the technological advances in steroid measurements and the assembly of detailed knowledge of steroidogenesis machinery and its expression in human fetal gonads. We summarise how the conversion of radiolabelled steroid precursors, antibody-based assays, mass spectrometry, ultrastructural studies, and the in situ labelling of proteins and mRNA have all provided complementary information. In this review, our discussion goes beyond the debate on recommendations concerning the best choice between the different available technologies, and their degrees of reproducibility and sensitivity. The available technologies and techniques can be used for different purposes and, as long as all quality controls are rigorously employed, the question is how to maximise the generation of robust, reproducible data on steroid hormones and their crucial roles in human fetal development and subsequent functions.

## 1. Introduction

The testes have long been considered as the conductor of the orchestra that is external male genitalia differentiation, thanks to the endocrine function relatively early during organogenesis. Indeed, the human fetal testes extensively synthesises both peptide hormones, such as anti-Mullerian Hormone (AMH), inhibin B, and insulin-like factor 3 (INSL3), and steroid hormones. This begins more or less as early as the endocrine somatic cells differentiate: Sertoli cells from the sixth post-conception week (PCW) (eighth gestation week (GW)) and Leydig cells from the seventh PCW (ninth GW), just after the presumptive gonad differentiates at the surface of the embryonic mesonephros.

The absence or alteration of endocrine function or unbalanced steroid production, inevitably results in congenital malformations that can have life-long outcomes for the male to be. These consequences are symptoms of testicular dysgenesis syndrome [1]. On the other hand, the major morphogenetic events taking place in female gonads during early steps of differentiation are often considered to be little more than the proliferation of germ cells that establishes the germ cell stockpile (ovarian reserve), which underpins fertility for the rest of the female’s reproductive life.

**Figure 1:**
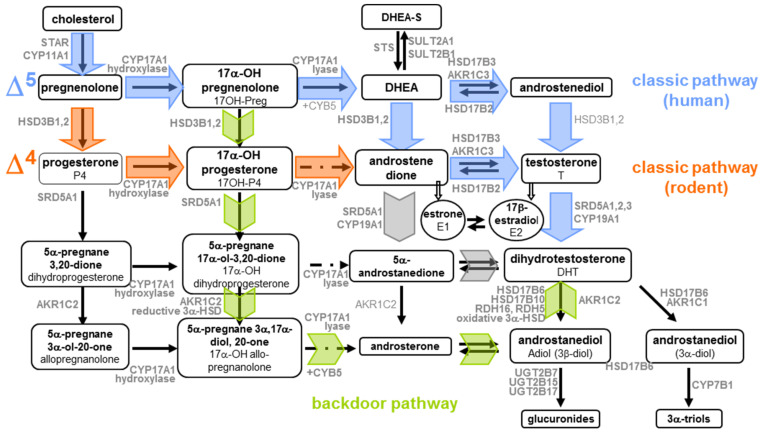
Summary of the principal gonadal steroidogenic pathways. Steroid precursors and metabolites from the classic delta 4 (orange arrows) and delta 5 pathways (blue arrows) are shown together with the backdoor (alternative) pathway (green arrows).

The conversion of cholesterol into the main biologically active reproductive steroids, testosterone (T), 5α-dihydro-testosterone (DHT), and 17β-estradiol (E2), can be achieved through several complex pathways (Figure 1, Supplementary Table S1). These involve numerous enzymes, including cytochromes P450 (CYPs) and hydroxysteroid dehydrogenases (HSDs) (Figure 1, Supplementary Table S2). In all cases in the male, steroidogenesis begins with the transport of cholesterol to the inner mitochondrial membrane of Leydig cells by transport proteins, such as STAR. In adult human testes, cholesterol is then preferentially converted into testosterone via the Δ5 pathway, using the following intermediates: pregnenolone (Preg), 17hydroxy-pregnenolone (17OH-Preg), dehydroepiandrosterone (DHEA), and androstenediol [2].

In parallel, there is a minor pathway, Δ4, which does not involve androstenediol as the last precursor to testosterone but rather androstenedione. Androstenedione can derive from the conversion of DHEA, and also from pregnenolone to progesterone (P4) and then to 17hydroxy-progesterone (17OH-P4). This Δ4 pathway is preferred in rodent gonads [3]. Finally, testosterone can be converted to a more active metabolite, DHT, which takes place in target tissues that express the enzyme 5α-Reductase 2, such as external genital tissues that require DHT for complete male sex differentiation [2]. Beyond these classical pathways of steroidogenesis, a third one, ending with the synthesis of DHT and bypassing testosterone, was characterised in testes from tammar wallaby pouch young and immature mice [4,5].

In human fetal gonads, the question of the preferred pathway is important to address because of the possible congenital abnormalities that could arise from mutations of genes encoding any step of steroidogenesis. These congenital malformations include cryptorchidism, which is a fault in testis descent along the abdomen and/or into the scrotum; hypospadias, which is abnormal development of the penis that leaves the urethral meatus proximal to its normal glandular position anywhere along the penile shaft, scrotum, or perineum; and micropenis. Since testicular androgens are involved in this masculinisation of the body, a better knowledge of the steroidogenesis pathway occurring at early fetal development is of crucial importance.

Increasing the knowledge of human fetal steroidogenesis should not be limited to basic science questions only since there are also significant clinical implications. Improving steroid detection in the maternal, fetal, and neonatal circulation; in amniotic fluid; and in the gonads, has applications in clinics, especially in the case of pathologies relating to disorders of differentiation and to congenital malformations. Furthermore, steroidogenesis has become a first-line screening endpoint in the field of endocrine disruption.

Indeed, many potential endocrine disrupting chemicals (EDCs) of current concern, identified by in silico or cell-line screening techniques, were originally assayed for their properties in more complex models, such as organotypic cultures of first trimester testes. Similar cultures have been performed for decades for human fetal ovaries; however, their endocrine function has not been well addressed. Overall, methodologies have to be compatible with several matrices, including blood, tissue explants, and culture medium.

In this review, we explore the history of steroidogenesis knowledge that has been acquired in lockstep with the development of increasingly sophisticated technological approaches. Each step improved our understanding of the complexity of fetal endocrine function in differentiating gonads and their relationships with other developing organs early during development. A better understanding of the steroidogenic function of gonads is critical far beyond research imperatives and is fundamental in the clinical setting, as well as for regulatory issues, including risk assessment and hazard identification, as well as improved accuracy in the prediction of the endocrine-disrupting properties of chemicals.

## 2. Histological and Ultrastructural Features of Steroidogenic Cells during Human Gonad Development in the Spotlight

**Figure 2:**
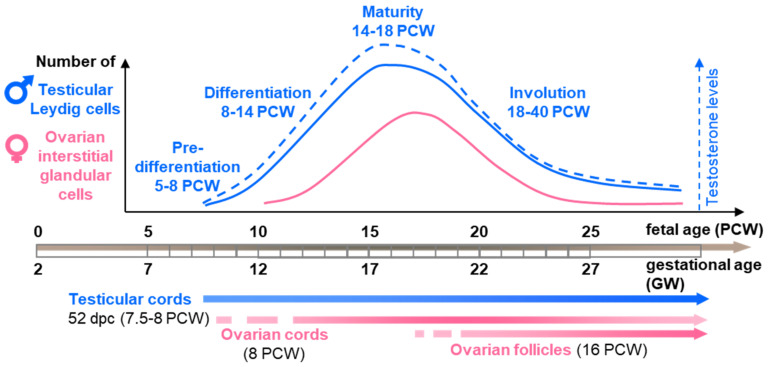
The dynamic progression of steroidogenic cell populations in the human fetal testes and ovaries. Profiles of the numbers of steroidogenic testicular Leydig cells (blue line) and ovarian interstitial glandular cells (pink lines) are depicted in comparison with the differentiation of testicular and ovarian cords, respectively, and of testosterone circulating profiles (adapted from [6,7,8,9,10]). Post-conception week (PCW); Gestational week (GW); and days post conception (dpc).

Gonads form as a thickening of the mesonephros during the fifth PCW (seventh GW) and are rapidly populated by germ cells. The first signs of histological dimorphism in the structure of differentiating gonads appear during the late sixth week as a clustering of somatic epithelial cells, the Sertoli cells, embracing germ cells into testicular cords. Ovarian cords (ovigerous cords) also form by the intrusion of the surface coelomic epithelium toward the interior of the gonadal territory, and epithelial pre-granulosa cells embrace the actively proliferating oogonia. However, these are only clearly distinguishable later, during the ninth week of development (Figure 2) [6,7].

The identification of Leydig cells in the fetal testis was delayed by more than a century compared to the first description of Leydig cells by Franz Leydig in adult testes in 1850 [11,12]. Classical histochemical detection of lipids in tissue sections showed complex lipid patterns in clusters of cells with a glandular appearance in the interstitium of human fetal testes in the 15th and 16th PCW (17th–18th GW), strikingly similar to those characteristics of mature steroid gland cells [13]. These Leydig cells display a diffusely distributed sudanophilic substance that develops throughout the cytoplasm, including the large Golgi zone, and contrasts with the cytoplasm of undifferentiated mesenchymal cells.

Histochemical techniques further highlighted that the diffuse lipids present in the Leydig cells were lipoproteins; however, no cholesterol could be demonstrated [13]. This was consistent with the structural role of lipoproteins in the membranes of the smooth endoplasmic reticulum where enzymes that are involved in the biosynthesis of hormone steroids reside. Immunostaining of testosterone and stereological quantification showed that Leydig cell numbers peak between the 15th to 16th PCW (17th–18th GW) and then decline progressively from the 22nd PCW (24th GW) up to birth and beyond, until the eighth month of postnatal age [9,10,14]. Nevertheless, despite this massive regression in numbers, some Leydig cells with typical fetal characteristics can still be identified in the testes for a few months after birth [9].

At the ultrastructural level, fetal Leydig cells displaying early features of steroid-secreting cells can be observed among the undifferentiated mesenchymal cells as early as the seventh PCW [10,15,16]. Their cytoplasm contains many polymorphic mitochondria, tubular smooth endoplasmic reticulum, some electron dense membrane-bound bodies, and few lipid droplets [10]. They mature by the end of the ninth PCW, grow larger, gather into clusters, and their number increases considerably [10,15]. Electron microscopy observations also demonstrated a basement membrane surrounding the Leydig cell clusters in human fetal testes [17].

Several cell types coexist in the fetal human testicular interstitium: fibroblast-like and myofibroblast-like cells, fetal Leydig cells, and degenerating Leydig cells. The latter display a dark nucleus and an electron-dense cytoplasm containing scant swollen smooth endoplasmic reticulum, residual bodies, and large lipid droplets [9,10]. This involution is associated with apoptosis [18]. Finally, a population of what are classed as “infantile” Leydig cells, containing an irregular infolded nuclei and the cytoplasmic accumulation of smooth endoplasmic reticulum and lipid droplets, are found in late fetal and neonatal testes [9].

Mitochondria always exhibit parallel cristae, even in the areas with abundant smooth endoplasmic reticulum [9]. Fetal Leydig cells appear to be the most abundant population, while low numbers of degenerating Leydig cells can be found at all ages. The numbers of infantile Leydig cells are initially low but increase progressively up to birth, remaining unchanged up to the second month of postnatal life [9].

In the second trimester human fetal ovary, a growing number of mitotic large, round, or ellipsoid cells of 10–30 µm in diameter are also grouped in clusters in the interstitial tissue close to blood vessels [6,7,19]. By the 13th PCW (15th GW), these interstitial type cells are located mainly around the ovigerous cords in the inner part of the cortex, while the outer half remain undivided by medullary fibrovascular tissue. The density of these cells peaks when the first primordial follicles form in the innermost region of the cortex but are not seen close to these follicles. After the 19th PCW (21rst GW), these cells decrease in number and almost completely disappear by the 38th PCW (40th GW) [7].

At the ultrastructural level, these round cells are observed as early as the 10th PCW (12th GW); however, they do not display the typical features of steroid-producing cells at this stage. They contain an accumulation of lipids, abundant smooth endoplasmic reticulum, large spherical mitochondria, free ribosomes, and well-developed Golgi apparatus [8,19]. At 13th–16th PCW (15th–18th GW), these round interstitial cells display a circular nuclear profile with dispersed chromatin and typical steroid-producing features, including abundant cytoplasm containing well developed agranular endoplasmic reticulum, large mitochondria with tubular cristae, and lysosomes [8,19]. These interstitial glandular cells also contain lipofuscin granules and stacks of annulate lamellae in their cytoplasm [20]. Moreover, intermediate cells with features between those of the fibroblast-like cells and interstitial cells are also present, suggesting that round interstitial cells might differentiate from fibroblast-like cells in the medulla [20].

## 3. 1960s–1970s: Conversion of Radioactive Precursors

### 3.1. Principle

The aim of this method is to highlight the metabolic pathways of synthesis or degradation in which a precursor of interest is involved. This method is based on the use of radiolabelled precursors created by synthesis. This is one of the most effective ways of understanding the fate of a molecule: the evaluation of metabolites that have inherited radioactivity makes it possible to conclude on the fate (short or long term) of the initial precursor and in which metabolic pathways it is involved. Under ideal conditions for their incorporation, these radiolabelled precursors are incubated in vitro, with the biological sample (whole organ, explants, tissues, or isolated cells) that expresses the enzymes necessary to metabolise this precursor. Unquestionably, the use of exogenous radioactive precursors has enabled a better understanding of what enzymatic activities the fetal gonads have and at what stages they are active.

### 3.2. Methodology

The first step is the incubation of the sample with a precursor of interest, which has either a hydrogen or a carbon atom substituted with radioactive isotopes, such as tritium (^3^H) or carbon-14 (^14^C), respectively. These are the two isotopes most suitable for the labelling of small molecules. Each of them has their own distinctive advantages and disadvantages [21]. For example, ^3^H has a better specific activity, and it is easier to manage in waste remediation due to its shorter half-life. However, ^14^C presents less potential for label loss. Moreover, ^3^H-labelled compounds are more easily synthesised than ^14^C-labelled compounds [22].

This may explain the slightly higher use of ^3^H-labelled steroid precursors. However, both isotopes were suitable for the study of steroidogenesis in the fetal gonads. Radiolabeled precursors are designed in such a way that their structure and activity are not strongly modified. The choice of precursor depends on the objectives of each study. For example, two precursors can be used and compared to determine the pathway that takes advantage over the other to lead to the synthesis of the same metabolite ([^3^H]testosterone and [^3^H]P4 for the study of the synthesis of 5α-Androstane-3α,17β-diol). This can also be [7α-^3^H]Preg.

The design of radiolabelled steroid precursors has to consider how steroid metabolites are modified through a sequence of chemical reactions. For example, the tritium radiolabelling of the hydrogen atoms bound to the pregnenolone-C-22 (22-[^3^H]Preg) is useless because these hydrogens are eliminated by the action of CYP17A1. Thus, the radiolabelling would be quickly lost after the transformation of 17OH-Preg into DHEA, and the study of the next intermediates and final products would be rendered impossible. For this reason, [7α-^3^H]Preg, or [7α-^3^H]P4 are commonly used, because these hydrogens are not modified during steroidogenesis.

Samples and precursors are then incubated, usually for 2 h, at 37 °C, with stirring and in a controlled atmosphere (95% O2 and 5% CO2). The buffer frequently used is Krebs-Ringer-phosphate-glucose pH 7.4. The reaction is stopped with chloroform:methanol (2:1), or acetone, and then the radioactive metabolites are extracted (with ethyl acetate or ethanol) and re-dissolved in a mixture of chloroform:methanol (2:1) [23,24]. The radioactive metabolites produced by the precursor during this incubation are then quantified.

Several methods exist to isolate these metabolites. Generally, thin layer chromatography is carried out. The solvents used as eluents vary from one protocol to another, including benzene:formamide, chloroform:methanol, and benzene:heptane:methanol:water. Very often, to help to identify the radiolabelled metabolites of interest, matching non-radioactive isotopes, known as “trainers”, are added to the extracts resulting from the incubation before being deposited on the plates. The silica plates are then dried after elution.

The metabolites are subsequently isolated and located on the plate by various methods: they can be visualised by spraying the plates with anisaldehyde or by observing them under UV light. The plates are then cut according to the observations made in order to definitively isolate each metabolite. Finally, the radioactivity is measured using a scintillation counter. This makes it possible to quantify the radioactive metabolites in pmol/mg of testis/2 h of incubation. To confirm the identification of the metabolites, recrystallisation with constant specific activity is sometimes carried out. Metabolites can also be separated and quantified by high performance liquid chromatography (HPLC) associated with a flow radio-detector.

The challenge of the radioactive precursor method is to identify and quantify metabolites that are similar and sometimes differ in function. Protocols must, therefore, include key steps to achieve good specificity. After the incubation of the tissues with the radioactive precursors, the steroids are extracted and supplemented by known amounts of radiolabelled standard corresponding to the targeted metabolites. These extracts are chromatographed on a celite column, and the metabolites are eluted by different solvent systems according to their typical elution profiles.

Recovery standards enable the peaks of the different metabolites to be clearly identified and distinguished. For example, estrone, estrone acetate, estrone diacetate, and estradiol diacetate are purified by different ratios of dichloromethane-ethyl-ether [25]. Sometimes, solvent systems do not allow the correct separation of metabolites [24]. In this case, chromatographic fractions are pooled and separated by thin layer chromatography with carrier steroids.

### 3.3. Conversion of Steroids in Human Fetal Gonads

Briefly, radiolabelled P4 and pregnenolone have been used as precursors to identify the metabolites generated by gonadal extracts in most of the studies published. Whole foetuses, minced organs, gonadal extracts, or even microsomes were used to decipher the functionality of the steroidogenic pathway (Table 1).

In testes, radiolabelled pregnenolone was metabolised into DHEA and T [23,24], while radiolabelled P4 was metabolised into 17α-OH-P4, 16α-hydroxyprogesterone, androstenedione, and T [24,26,30,38]. 17β-hydroxy-5α-androstan-3-one and 5α-androstane-3α,17β-diol were also found [34]. Pregnenolone sulphate was metabolised into DHEA, androstenedione, and T [31]. The metabolism of pregnenolone into androst-5-ene-3α,17β-diol and 3β-hydroxyandrost-5-ene-17-one suggested that mid-term human fetal testes displayed 5 α-reductase activity [34]. In addition, radiolabelled androstenedione can be converted into T [38]. Altogether, these experiments suggested that the enzymatic machinery, including CYP17A1, HSD17B, and HSD3B enzymes, was active in human fetal testes as early as the first trimester of pregnancy.

Further comparison of the rates of conversion of 17OH-Preg and 17OH-P4 by fetal testis microsomes into DHEA and androstenedione, respectively, indicated that the delta 5 pathway of CYP17A1 yielded 11-fold higher levels metabolites than did the delta 4 pathway [36]. In first trimester testes, deuterated 17OH-P4 can be converted into androstenedione, T and 5α-17hydroxy-pregnanolone via both the classical and alternative pathways. The presence of active alternative pathways bypassing T was confirmed by the incubation of four different steroids along the pathway (and the detection of the relevant metabolites (Table 1) [37].

The use of radiolabelled sodium acetate allowed a broader screening of the pathway in testes with the identification of the C21 steroids 3β-hydroxy-Δ5-pregnen-20-one, 17-hydroxy-Δ4-pregnene-3,20-dione, and P4, in addition to the C19 steroids Δ4-androstene-3,17-dione, and T [28,29]. All studies tended to show that steroidogenesis in the human fetal testis led to the production of T, widely regarded as the effective testicular androgen. This production coincides directly with the morphological differentiation of Leydig cells.

The quantification of 17OH-Preg inherited from radiolabeled pregnenolone showed that the quantity of this metabolite increased as T production decreased in foetuses around 19 PCW [24]. The accumulation of this T precursor leads to a hypothesis concerning the regulation of T production. Specifically, the activities of enzymes used to transform 17OH-Preg into T are inhibited. These results were consistent with the observation of a decrease in C17-C20 hydroxylase (which allows the formation of DHEA from 17OH-Preg) activity in foetuses of 19–20 weeks [31].

Similar investigations of the human fetal ovary showed that it could efficiently convert radiolabelled P4 into 20α-hydroxy-4-pregnene- 3-one, from 19 weeks onwards, but neither T nor androstenedione were detectable [24,26,27,30]. Using radiolabelled pregnenolone, research identified P4, 17OH-Preg, 5-pregnene-3β,20α-diol, 5-pregnene-3β,17α,20α-triol, 5α-pregnane-3,20-dione, and DHEA as the resulting metabolites, suggesting efficient 5-ene-3β-HSD, 17-hydroxylase 20α-HSD and 5α-reductase activities [32]. Again, no T was found in these experiments [24,33]. Finally, the conversion of radiolabelled T and androstenedione into estrone and E2 (by the 7th–8th PCW [9th–10th GW]) by human fetal ovaries suggested efficient CYP19A1 aromatase activity [25].

More recently, the incubations of first trimester ovaries with deuterated 17OH-P4 led to the identification of androgens from both the classical and alternative pathway, specifically, androstenedione and 5α-17hydroxy-pregnanolone, respectively. Incubations with radiolabelled 5α-17-hydroxy-pregnanolone, 5α-androsterone, and 5α-androstanediol were associated with the detection of several androgens from the alternative synthesis pathway, indicating that this pathway is active in the human fetal ovaries (Table 1) [37].

While this approach paved the way to a better understanding of the functionality of the steroidogenic machinery in human fetal gonads and allowed the determination of steroid concentrations as low as the picomole range, it displays several limitations. First, it uses radioactive elements that require compliance with strict laboratory safety rules and complications of waste disposal. Second, some precursors are not available in their radiolabelled form, and the recrystallisation steps required to characterise radiolabelled metabolites are extremely time-consuming. Third, the numerous studies using this approach showed a lack of standardisation.

These differences included the nature of the precursor, the choice between hydrogen or carbon for labelling, the separation of the metabolites, and the measure of their radioactivity. Finally, the units of measurement greatly differed from one study to another. For instance, in the case of testosterone from radiolabelled precursors, some results were given in pmoles/10 mg of testes/2 h, or pmoles/pair of testes/2 h [24]. Other units were used, such as pmoles/mg of protein [31], or the conversion percentage of the radiolabeled precursor [23]. This difference in the presentation of results from one study to another complicates the comparisons and the interpretations.

Overall, these studies showed that many enzymes of the steroidogenic pathway are active from the early steps of gonad differentiation in both testes and ovaries.

## 4. Advent of Antibody-Based Assays

### 4.1. Principle

The so-called “immunological” methods are based on the formation of immune complexes between an antibody and its (hopefully) specific antigen. These methods are often used for the detection and quantification of antibodies or blood antigens in the pico-gram/mol range. Once monoclonal antibodies against a molecule of interest are available, these bioassays could be adapted to other targets that go beyond the purely immunological aspect. In contrast to conversion-based assays, antibody-based assays directly measure a target, independent of any radiolabelled steroid. Therefore, these methods differ in the nature of the signal measured. In addition, antibody-based assays allowed for a broader analysis of the system since it is possible to use them to measure steroids in different matrices, such as the plasma, as well as in organ extracts, and medium from cultured organs.

### 4.2. Methodologies

**Figure 3:**
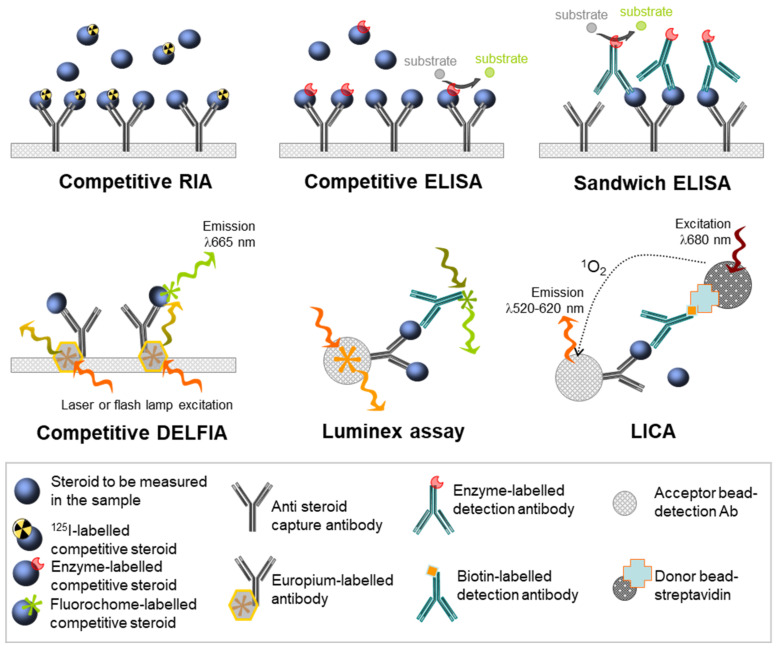
A schematic explanation of the principal immunological assay types. All the immunological methods represented here rely on the use of a steroid-directed specific detection antibody fixed on a solid phase or on beads. The main difference lies in the detection technique. The steroid of interest-antibody complex will be detected even by a radioactive competitor (radioimmunoassay; RIA), or an enzymatic reaction (Enzyme Linked ImmunoSorbent Assay; ELISA), fluorescent dyes (Dissociation-enhanced lanthanide fluorescence immunoassay; DELFIA, Luminex technology), or chemiluminescent dyes (light-initiated chemiluminescent assays; LICA). The main components for each technology are described in the box.

Detection antibodies are coupled with a system of detection, which allows the visualisation and then the quantification of the immune complexes formed. The main dichotomy between immunological assays lies in the use of either radioactive or non-radioactive detection systems (Figure 3).

Radioimmunoassays (RIA) are sensitive due to the use of radioelement bound antibodies for the detection of steroids. The quantification of T by RIA can be preceded by an extraction step using an organic solvent. The assay is indirect if this extraction step takes place. Extraction removes the matrix and, in particular, the proteins interacting with T [39]. This extraction is generally associated with a chromatography step to isolate the T from the other metabolites whose structure is similar to T, and limit the cross-reaction phenomenon, leading to an overestimation of the concentrations [40,41]. These preliminary steps are mandatory for the study of adult testes steroids because the medium used for their culture is more complex.

On the other hand, these steps are costly, time-consuming, add an additional step with an inherent increase in the coefficients of variation, and require a high sample volume. Therefore, direct quantification without extraction has many practical advantages. It has been validated for the study of secretions from the fetal testes, although, in some situations, this can lead to interference problems caused by other steroids. For instance, RIA against T for human fetal testis culture media was derived from the method used for rat testes [42,43].

The antibody used displayed cross reactions with DHT and, to a lesser degree, with 5α-androstane-3β,17β-diol. However, extraction or prior chromatography was not required because DHT is secreted in minute amounts by the rat fetal testes [42,43]. Sometimes, results were reported as the sum of T and 17β-hydroxy-5α-androstan-3-one [43]. It is, therefore, important, depending on the experimental model, to validate and compare the results obtained with or without preliminary extraction.

The commercially available steroid RIA kits use the competition between the antigen in the sample and fixed amounts of the same antigen labelled with ^125^I isotope. Culture media are incubated in plates coated with anti-testosterone antibodies, together with ^125^I-T of known concentration. Both types of T compete with each other and create immune complexes with the attached detection antibodies: the more unlabelled T, the lower the measured radioactivity. The radioactivity is measured using a gamma scintillation counter that measures the number of counts detected per minute (cpm). Quantification is performed using a standard curve allowing for correspondence between the concentration of unlabelled T and the radioactivity measured. Although sensitive, this method has major drawbacks, such as the handling of radioactivity and its low throughput rates.

Several non-radioactive antibody-based methods have been developed, with gradual steps of improvement. Historically, the enzyme-linked immunosorbent assays (ELISA) were broadly used for rodent samples, with the main advantage being the removal of the need for radioactivity. Variations in ELISA methods include direct, competitive, antibody-sandwich, and double antibody-sandwich ELISAs (Figure 3). In direct ELISA assays, the specific detection antibody is linked to an enzyme, which, in the presence of its substrate, produces a colored reaction.

The intensity of this chromatic change, measured by spectrophotometry, is proportional to the quantity of antibody-enzyme couples that have reacted with the specific antigen. Several ELISA kits have been developed for the determination of T, although this hormone is generally quantified using RIA [44]. In competitive ELISAs, labelled antigens of known quantity and non-labelled antigens from the samples compete for binding to the antibodies fixed on a support in limited quantities. Competitive antigens are often labelled with an enzyme such as acetylcholinesterase. Then, the greater the number of antigens to be assayed, the greater their proportion among the antigens retained by the antibodies, and the weaker the signal will be.

Most commercially available ELISA kits use this competitive technology, likely because this assay needs only one highly specific antibody. However, most steroid-quantification ELISA kits use the so-called competition method. The “sandwich” ELISA, which is designed for the detection of soluble antigens, is based on the capture of the antigen between two antibodies directed against distinct epitopes of a single antigen of interest: the capture antibody, which is fixed on the support, and the detection enzyme-linked antibody, which recognizes the protein/first antibody complex (Figure 3).

Increasing staining indicates an increasing concentration of antigens. If the detection antibody is not conjugated to the enzyme, a second detection antibody is required, and this is referred to as a double sandwich ELISA. These sandwich methods increase the sensitivity and specificity because two antibodies specific to the antigen are used. However, it can be difficult to optimise these two antibodies or even to obtain them, especially when the target antigen is a molecule with a low molecular weight, such as steroids.

Both RIA and ELISA immunological methods are very accessible, and due to the development of ready-to-use kits, it is even possible to automate ELISA for significant repeatability. The major drawback of RIA and ELISA immunoassays is their specificity for a single antigen of interest, which entails a certain cost, not only in terms of price but also in terms of sample. When the goal of a study is to quantify a large panel of molecules, it is necessary to take as many samples as there are molecules to be assayed.

This constraint can be overcome if these molecules are secreted in large quantities or if the kits used are highly sensitive, thus, allowing a lower sample volume for quantification. If a molecule is secreted in a small quantity, then assays consume a large volume of valuable medium. The perpetual developments of technologies have more recently made it possible to drastically reduce two major drawbacks of this approach: increases in the power of the detection system in order to increase the sensitivity, the possibility of multiplexing the measurements, and miniaturization—allowing for a lower sample input size. During the last decade, improvements of antibody-based assays focused on the detection systems.

The DELFIA (dissociation-enhanced lanthanide fluorescence immunoassay) technology (from Perkin Elmer) uses the fluorescence resonance energy transfer (FRET) principle associated with Homogeneous Time Resolved Fluorescence (HTRF) ligands. The conjugate associated with one antibody against the target excites the conjugates associated with the competitor ligand or with another specific antibody [45,46]. The properties of lanthanide chelate labels, including an intense long-lived fluorescence and a large stokes shift, contribute to an increased signal-to-noise ratio and minimize the effect of autofluorescence of samples (Figure 3).

The DELFIA technology was developed for the detection of various analytes in different biological matrices. The workflow of the technique includes a short incubation of the sample with an antibody-coated microplate, followed by the addition of the lanthanides-labeled detection antibody. The chelate is then released by the addition of an enhancement solution, allowing a new highly fluorescent chelate. The amount of analyte is proportional to the emission signal, which can be quantified by interpolation from a standard curve.

While DELFIA technology did not improve the effectiveness to detect T, E2, and P4, compared with RIA technology, the efficiencies of the test performances were improved. Moreover, these kits avoid the need for solvent extraction, radioactivity, or scintillation cocktails [46]. In addition, multiplexing is possible due to different lanthanide chelates having their own narrow emission spectra, while labelled components can remain usable for a year—much longer than ^125^I-labelled components.

Luminex technology is based on microspheres that are internally labelled with graded proportions of a red and an orange dye, providing the capacity to identify each bead (up to 100 beads) [47,48]. Then, the principle is similar to ELISA: beads of a single identity are covalently coupled to a specific capture antibody for the analyte of interest. Once the analyte is fixed to the bead, a second detection antibody is used to quantify the amount of analyte captured.

This secondary antibody is either directly conjugated to the phycoerythrin (PE) fluorophore or biotin, which is then reacted with streptavidin-phycoerythrin. For the analysis step, the suspension passes across the path of two laser beams, allowing the simultaneous analysis of the different fluorochromes associated with the same bead. The first laser (635 nm) excites the internal orange and red dyes, which emit at two very different wavelengths to identify the bead and, therefore, the analyte. The second laser (532 nm) excites the fluorochrome (PE), which is fixed to the surface of the bead during the reaction, making it possible to quantify the analyte.

Other technologies were also established in order to achieve efficient and sensitive detection of hormones in human serum for use in routine diagnostics, or to push back the detection limits. Preconcentration methods using anti-E2 aptamer-anchored isothiocyanate-modified beads (NCS beads) were designed to overcome the very low concentrations of E2 in the environment [49]. Light-initiated chemiluminescent assays (LICA) set up for E2 detection are easy to automate and provide rapid analysis, compatible with clinical requirements [50]. E2 can also be detected at concentrations lower than 10 pg/mL in human serum by using surface-enhanced Raman scattering (SERS)-based immunoassay using functional nanomaterials [51]. The LICA and SERS-based technologies are both based on a competitive method with a labeled tracer biotinylated-BSA-E2/E3 and E2-nanoparticles conjugated, respectively.

The essential principle of LICA is the energy transfer of singlet oxygen atoms in the luminescent oxygen channeling immunoassay (LOCI), whereas the (SERS)-based immunoassay quantifies Raman signals adsorbed onto the nanoparticle surface. Yet, while these technologies began to be used for steroid quantifications in animal models, cell lines, and clinics, they have not been used in the case of human fetal gonads, with the exception of DELFIA technology, even though lowering the sample volume is currently an obvious way to better exploit the limited volumes of media available from organotypic cultures [45,46]. This may be due to their as yet insufficient sensitivity, for which the addition of extraction steps is still required [49,52]. These technologies require very costly equipment, which remains rare in most academic research laboratories.

Whatever the immunological method, however, the main problem with antibody-based methods is their dependence on the quality, and, more specifically, the specificity of the antibodies. Therefore, the issue of cross-reactions must be considered: an antibody specific to a given antigen can have an affinity for other molecules that are structurally similar to the target antigen and present in the sample. This may, therefore, overestimate the concentrations measured [41]. This issue is particularly evident when it comes to steroid assays. In the case of T, for example, the antibodies used often have a partial affinity for DHT, which is also secreted in vitro by the fetal testes (unpublished data).

### 4.3. Antibody-Based Assays for Human Fetal Gonadal Steroids

Immunoassay methods were mainly used to measure T from human fetal testes, leaving out other steroids, and ignoring the ovaries. First used to mirror measurements of T in the blood, in order to assess the dynamics between circulating and testicular production levels [53,54,55], these methods were used to investigate the roles of trophic hormones. This helped to demonstrate the importance of human chorionic gonadotrophin (hCG) and modalities of action for the stimulation of steroidogenesis in human fetal testis. This stimulation of T production by hCG varies with the age of the foetuses [54].

Fetal plasma concentrations (35 ng/mL) of hCG can stimulate T production in cultured testis [56]. A similar study showed that hCG failed to enhance T synthesis in human fetal testicular fragments [57]. To explain this discrepancy, the authors proposed the hypothesis that the hCG receptors might be already fully occupied by endogenous hCG. Unlike the adult testis, Leydig cell desensitisation by hCG does not occur in the human fetal testis [58]. Altogether, these studies not only improved the understanding of the physiology of the human fetal testis but also enhanced the methodology of organotypic cultures enabling a more realistic modelling of the physiological environment.

Similar methods were used to identify the appropriate trophic reagents to sustain human fetal ovaries in culture, showing the spontaneous production of DHEA, androstenedione, E1 and E2, and stimulation with Bu2cAMP [59]. The refinement of antibody specificities associated with chromatography fractionation allowed for a large-scale screening of seven different steroids from testes aging aged 4th–18th PCW (6th–20th GW): pregnenolone, P4, 17OH-P4, DHEA, androstenedione, T, and E2 [60].

More recently, the RIA method was more or less systematically used to measure T production from cultivated human fetal testes in reprotoxicology studies (Table 2). The validation of gonadotrophin supplementation to sustain T production, and germ cell viability [61] paved the way for a standardised methodology to cultivate testes and, then, expose them to a wide range of chemicals and radiation (Table 2). RIA was revealed to be sufficiently sensitive to detect alterations in T production despite the inter-individual variability characteristics of the human model.

However, some controversy remains. In the case of bisphenols, the question arose regarding whether testes have to be cultivated under basal conditions (without gonadotrophin supplementation) or in a manner closer to real-life physiological conditions (i.e., with gonadotrophin supplementation). Indeed, following exposure to low concentrations of BPA, a decreased T production was observed only in the absence of gonadotrophin supplementation [62,63,64,65] with concomitant uncertainty about the physiological relevance to humans.

Despite the wide range of T concentrations that can be produced by first trimester human fetal testes during differentiation in culture, RIA methods allow for the identification of windows of sensitivity, as exemplified by studies of the effects of ibuprofen. Indeed, the levels of T produced by 8–10 PCW (10–12 GW) testes can be altered by biologically-relevant concentrations of ibuprofen, unlike younger and older testes [66]. In the same study, data obtained in a large number of samples due to RIA were consistent with similar experiments measured by mass spectrometry. In toxicological studies on the human fetal testis, a focus is generally made on T and neglecting the precursors and metabolites.

**Table 2:** Steroid measurements in ex vivo studies. Pregnenolone (Preg); 17-hydroxy-pregnenolone (17OH-Preg); Progesterone (P4); 17-hydroxy-progesterone (17OH-P4); dehydroepiandrosterone (DHEA); androstenedione; testosterone (T); 5α-dihydrotestosterone (DHT); estrone (E1); human chorionic gonadotrophin (hCG); human luteinizing hormone (hLH); 22R-hydroxycholesterol (22R-OH), bisphenol (BP); seminal vesicle weight (SVW); reverse transcription-quantitative PCR (RT-QtPCR); immunohistochemistry (IHC); and gas chromatography–tandem mass spectrometry (GC/MS-MS).

Year	Technique	Chemical/Injury	Measurement Technique	Measured Steroids	Reference
1991	injection of hormones before abortion	Injection of norethindrone acetate and ethinyl estradiol before abortion	Conversion of radiolabeled DHEA	T, androstenedione	[67]
2006	culture	(−) Culture validation (+/− hLH) Retinoic acid	RIA RT-QtPCR (*CYP11A1*, *CYP17A1*, *STAR*)	T	[68]
2007	culture	Dieldrin +/− hLH	EIA DELFIA^®^/fluo IHC (STAR)	T	[69]
2007	culture	di(n-butyl) phthalate (DBP) monobutyl phthalate (MBP) +/− hCG, +/− 22R-OH	RIA	T	[70]
2007	culture	irradiation	RIA RT-QtPCR (*CYP11A1*, *CYP17A1*)	T	[71]
2009	culture	mono-2-ethylhexyl phthalate (MEHP) Ketoconazole	RIA RT-QtPCR (*CYP11A1*, *CYP17A1*, *STAR*)	T	[72]
2010	culture	Cadmium (+/− hCG)	RIA	T	[73]
2012	culture	Metformin	RIA	T	[74]
2012	xenografts	di-n-butyl phthalate (DBP) monobutyl phthalate (MBP)	RIA Seminal Vesicle weight (SVW) RT-QtPCR (*STAR*, *CYP11A1*)	T Androgen action	[75]
2012	xenografts	phthalates	RIA RT-QtPCR (*SCARB1*, *STAR*, *CYP11A1*, *CYP17A1*)		[76]
2012	culture	Bisphenol A Diethylstilbestrol	RIA	T	[62]
2013	culture	Paracetamol Aspirin Indomethacin	RIA	T	[77]
2013	xenografts	Diethylstilbestrol	RIA SVW	T Androgen action	[78]
2014	xenograft	abiraterone acetate di-n-butyl phthalate (DBP)	RIA Androgen sensitive organ weights	T, P4 Androgen action	[79]
2015	xenografts	Acetaminophen (paracetamol)	RIA SVW	T Androgen action	[80]
2015	culture	Bisphenol A (BPA), BPS, PBF	RIA	T	[64]
2017	culture xenografts	Ibuprofen	RIA GC-MS/MS RT-QtPCR (*STAR*, *BZRP*, *CYP11A1*, *CYP17A1*, *HSD17B3*, *SRD5A3*) RIA	T Endogenous: T, DHEA, Preg Produced: 17OH-Preg; DHEA; 17OH-P4; T; DHT androstenedione; T	[66]
2017	culture	27 chemicals (caffeine; ethanol; paraxanthine; theobromine; theophylline; 1,3,7 trimethyluric acid; atrazine; bitertanol; chlordecone; glyphosate; imazalil; orto-phenylphenol; prochloraz; propiconazole; aniline; BPA; BPB; BPE; BPF; BPS; aspirin; clomiphene; ibuprofen; indomethacine; ketoconazole; paracetamol; valproic acid)	RIA	T	[81]
2018	culture xenografts	Specific ALK4/5/7 inhibitor SB431542	LC-MS/MS RIA SVW	17OH-P4, T; DHEA; androstenedione; P4, E1 sulphate, T	[82]
2018	culture xenografts	BPA	RIA RT-QtPCR (*STAR*, (*CYP11A1*, *CYP17A1*, *CYP19A1*) SVW	T	[65]
2019	culture	recombinant FGF9 tyrosine kinase inhibitor SU5402	LC-MS/MS	P4; 17OH-P4; T; DHEA; androstenedione	[83]

To date, most studies on human fetal gonads have used RIA and none have used ELISA, to the best of our knowledge. Although routinely used in clinics, and while claimed to be more sensitive than ELISA, only DELFIA non-radioactive technology has, thus far, been used for the quantification of steroids from human fetal gonads [69]. It remains surprising that antibody-based technologies allowing for multiplexing and low-quantity sample volume have not been used so far in the field. Indeed, understanding the whole steroidogenic pathway is crucial in the context of the characterisation of action of possible endocrine disruptor, but is currently mostly addressed by other techniques.

The absence of data on the impact of chemicals regarding the steroidogenic capability of the human fetal ovary is an obvious and troubling data gap in the field but may be explained by the relatively low limit of detection and quantification of immunoassays in regard to the levels of the final steroids (E2) but not of the precursors [59].

## 5. Steroidogenic Fingerprints

In a way similar to the direct measurement of steroids, or indirect measurement of steroid enzyme activity, the ontogeny and cell specificity of expression of the genes for steroidogenic enzymes have been investigated at the mRNA and protein levels in human fetal gonads following the development of in situ techniques.

At the bulk mRNA level, both ovaries and testes have been investigated for the expression of *CYP11A1* (also known as P450 side chain cleavage enzyme) and *CYP17A1* (17 alpha-hydroxylase/17,20-lyase), both of which are expressed in the gonads [84]. Fluctuations of expression of mRNA and proteins, peaking at 8–12 PCW for the testes and the later but constant expression in the ovaries, correlated well with the respective steroid profiles [14,84,85,86]. In addition, even low levels of expression of mRNAs representative of the steroidogenic machinery in several non-endocrine tissues from second trimester foetuses (13th–17th PCW [15th–19th GW]) suggested that they could be sufficient to produce local (i.e., autocrine and paracrine) effects [86].

In situ, the immunolocalization of testosterone allowed the identification and quantification of testosterone-producing cells [9]. Immunohistochemistry and/or in situ hybridisation for the steroidogenic acute regulatory protein (STAR), which mediates the translocation of cholesterol from the outer to the inner mitochondrial membranes, 3beta-hydroxysteroid dehydrogenase (HSD3B) and CYP17A1, and its modulator cytochrome b5 (CYB5), in testes were consistent with Leydig cell expression [87,88,89]. CYP19A1 was found simultaneously in Sertoli, Leydig, and germ cells, especially between 13 and 22–24 PCW with mRNA profiles consistent with protein expression [90]. Image analysis of CYP17A1 immunostaining showed an increase in the proliferating cell nuclear antigen-positive interstitial cells during Leydig cell hyperplasia [88].

Consistently, parallel stereological quantification of the Leydig cell population and quantification of mRNA levels of genes encoding Leydig cell markers (*HSD17B3*, *CYP11A1*, *PTC1*, *CYP17*, *LHR*, and *INSL3*) showed that the levels of transcripts remained static per cell [14]. Discordant patterns of expression were found for estrogen receptor beta (*ESR2*) in the developing testis [90,91]; however, this may rely on the quality of antibodies directed against estrogen receptors [92]. Androgen receptor (AR) was detected in undifferentiated testicular cells, peritubular myoid cells, and in some Leydig and stromal cells from five PCW (seven GW), with a continuous increase of the number of AR positive stromal cells through to 20 PCW (22 GW) [91].

In the ovaries, intense staining of STAR was found in sporadic stromal ovarian cells particularly in the hilar region at 17 PCW (19 GW) and older [87]. While no staining for CYP17A1 or CYB5A was found in mid-gestational ovaries [89], another study led to the classification of three populations of CYP17A1-expressing interstitial cells in the second trimester ovaries: (i) primary interstitial cells, located between the ovigerous cords near the cortical-medullary border where meiosis and primordial follicle formation occur, (ii) a few positive cells between 25 and 32 weeks, and (iii) theca interstitial cells surrounding developing follicles observed after 33 weeks [93]. Morphometric analysis revealed a progressive decrease in the number of primary interstitial cells during the second trimester, suggesting that primary interstitial cells might have a finite lifetime.

The characterisation of key members of the steroid-signalling pathway that are expressed in the second trimester human fetal ovaries showed a continuous increase in mRNA levels encoding the steroidogenic apparatus and steroid receptors [94]. In situ analysis showed immunostaining for CYP19A in pre-granulosa cells around primordial follicles and somatic cells around oocyte nests and CYP11A1 in some pre-granulosa cells [94]. Different studies reported the expression of proteins of the steroidogenic machinery in oocytes: STAR at 30 PCW (32 GW), and CYP11A1, CYP17A1, and HSD3B2 during the second trimester [87,94].

While it could be hypothesised from STAR expression that STAR may have roles in metabolic processes in addition to stimulating pregnenolone synthesis [87], the concomitant expression of several other enzymes suggests that the oocyte may contribute to steroidogenesis in the human fetal ovary [94]. Receptors for steroids, on the other hand, are also expressed in the human fetal ovaries, with ESR2 localised primarily to germ cells, and AR exclusively in somatic cells [94], thus, indicating that, at least at the time of follicle formation, human fetal ovaries express the machinery to produce and detect multiple steroid signaling pathways. The unexpected profiles of enzyme expression in oocytes demonstrates distinct species specificity compared with rodents.

Several non-targeted bulk analyses of human fetal gonad transcriptomes using different technologies have been published [95,96,97], and genes encoding enzymes of the steroidogenesis pathway have been used as Leydig cell markers. However, deep analyses of the numerous isoforms and sexual comparative analysis remain in the to-do list. The current race to single cell and/or spatial RNAseq analysis may also, in the near future, provide a better understanding of the dynamics of the expression of proteins of the steroidogenic pathway and of their possible isoform specificities and may highlight cell specificities in the steps of cell lineage [98,99].

Single cell RNAseq allowed the tracing of the lineage of interstitial progenitors for Leydig cells from the early steps of differentiation up to postnatal life. The identification of markers for each step of differentiation and of species specificities compared with mice [99] also resulted; however, this study was focused on the testis, leaving the ovary in a state of comparative darkness in this respect.

In toxicological studies, surveys of the expression levels of proteins of the steroidogenic machinery by quantitative PCR have also been used as endpoints, with STAR, CYP11A, and CYP17A1 being the most studied (Table 2). Of note, several xenobiotics induced a concomitant alteration of testosterone production ex vivo as well as the expression of these proteins [66,72,75]. However, alterations in steroid production may not be systematically associated with a decrease in the expression of specific steroidogenic enzymes. Examples include cases where the mechanism(s) of action of the xenobiotics target the function(s) of the enzyme(s) as described for anti-epileptic drugs and isoflavone derivatives [100,101,102].

## 6. XXIth Century and Mass Spectrometry

### 6.1. Principle

Mass spectrometry (MS) is an analytical method that allows the identification and, usually, the quantification of molecules through the direct analysis of their mass to charge ratio. This avoids the risk of cross-reactivity that is observed with RIA or ELISA. Today, MS is suitable for the study of molecules of all sizes, such as proteins and macromolecules, including small molecules, such as steroids.

The spectrometer is typically coupled to a chromatographic system (usually gas chromatography (GC) or liquid chromatography (LC)). LC and GC platforms allow the separation of molecules before MS analyses (including those with the same masses that can potentially give similar fragments) according to their physico-chemical properties (e.g., hydrophobicity). Then, ionisation in the source before the molecules enter the MS analyser allows the attribution of positive or negative charges to the molecules. Subsequently, the goal is to separate these ionised molecules according to their mass to charge ratio (*m*/*z*) with an analyzer. This data is then processed and represented in a mass chromatogram.

### 6.2. Methodologies

There are many technologies that allow the separation and ionisation of molecules, and there is a wide variety of analyser types (quadrupole, orbitrap, time of flight [TOF], etc.), with different properties and advantages (e.g., mass accuracy, spectral resolution, velocity, and sensitivity). It is essential to select the right technology based on the physico-chemical properties of the molecules and, in some cases, the matrices, to ensure good selectivity and sensitivity. Tandem mass spectrometry (MS/MS) makes it possible to combine two analysers, in order to take advantage of the benefits of each, to achieve more selectivity (e.g., the selection of a precursor followed by the selection of its fragments using low or high resolution).

**Figure 4:**
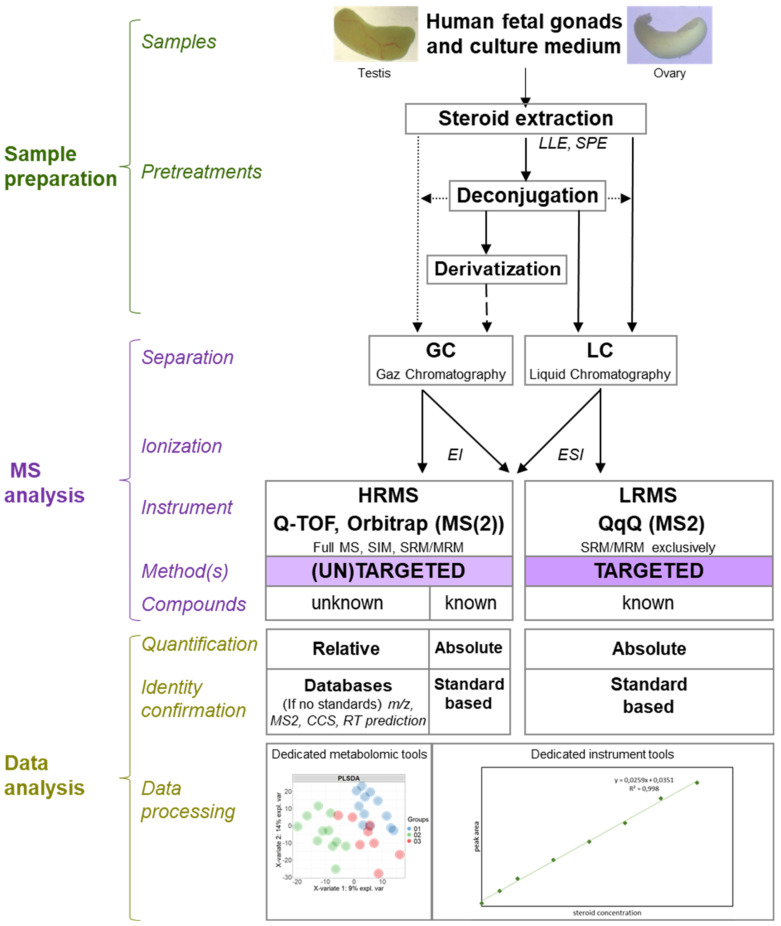
Steroidomics workflow. Mass spectrometry analysis of steroids requires preliminary steps for the sample preparation. Steroids from human fetal gonads or culture medium are first extracted by liquid–liquid extraction (LLE) or solid-phase extraction (SPE). This step allows the concentration of the steroids and reduction of the biological matrix. The subsequent deconjugation and derivatization steps are necessary to, respectively, release the free steroid if needed and obtain chemical properties useful before the mass spectrometry analysis: good volatility, heat-resistance, and ionizability. Samples are then injected through the chromatographer (LC or GC) mounted upstream of the mass spectrometer to separate all steroids. The ionisation step (EI or ESI) transforms neutral steroids into mono or multicharged ions (z > 1), but only monocharged ions are analysed next. The sample preparation protocol and the ionisation method used depend on the chromatographic method. Then, ionised steroids are analysed according to their mass to charge ratio (*m*/*z*) by the tandem mass spectrometer analysers (MS2). Tandem mass spectrometers suitable for the study of steroids are Quadrupole-Time of Flight (Q-TOF), Q-Orbitrap, and Triple Quadrupole (QqQ). Due to their performances (high mass resolution, mass accuracy, and dynamic range) Q-TOF and Q-Orbitrap are mostly used in full scan mode and for non-targeted steroid profiling. In contrast, the QqQ instrument, a low-resolution mass analyser is used for targeted approaches. The final step of data processing depends on the tandem analysers used and the approach chosen (untargeted or targeted). Quantitative determination is absolute or relative, and steroids are identified following either database searches or comparison against high purity standards.

Due to this enhanced selectivity, MS/MS is ideally suited to the analysis of complex biological samples containing very similar molecules, sometimes isobaric as is the case for T and DHEA or for DHT and androstenediol. Ion mobility also makes it possible to discriminate isobaric molecules, including steroids, by using inert gases to separate isobaric molecules according to their different conformations and groupings [103]. In the case of the study of steroids, and more precisely their quantification, several types of tandem mass spectrometer are used (Figure 4). Moreover, it is often a question of comparing their performances, in terms of the sensitivity, specificity, resolution, etc. [104].

The methods for extracting and concentrating the molecules of interest are often the subject of method developments. It is essential to compare their efficiencies for accurate extraction of the molecule(s) of interest and efficient elimination of the components of biological matrices (e.g., plasma, urine, or even saliva) at the same time [105,106]. The, sometimes minute, quantities of certain steroids require sufficient sensitivity to ensure detection in the range of pg/mL [104]. Steroid extraction is often performed by liquid–liquid extraction (LLE) between organic and aqueous phases.

This method is particularly simple and quick to set up. Other techniques, such as solid phase extraction (SPE) using columns, can be employed on their own or after LLE or protein precipitation using organic solvents [106]. LLE and SPE using reversed phase sorbents are ideal for extracting low abundant steroids [107]. The LLE method is appreciated for its speed and simplicity, while the SPE method can be semi-automated using 96-well plates for instance [106]. The quality of the extraction technique used is evaluated by the calculation of the percentage of steroid recovery (% of recovery), precision, and accuracy as well as the matrix effect (%) [108].

The presence of chromatography, upstream of the spectrometer, is particularly necessary in the case of the analyses of steroids. The chromatographic retention time is used as a criterion to support the identification of steroids and can allow the separation of isobaric steroids. The use of GC requires a derivatisation step before the injection of extracts. Derivatisation makes steroids less heat-sensitive and more volatile: two aspects necessary for separation by GC.

On the other hand, derivatisation renders steroids more easily ionisable, thus, increasing the overall sensitivity that can be achieved [109]. Over the last ten years or so, GC-MS/MS has gradually been replaced by LC-MS/MS for the study of metabolites, including steroids [107]. The preferred use of LC reflects an improvement in the separation performance of these chromatographic systems [106]. Indeed, the matrix effects of the samples can cause considerable interference during LC-MS analyses.

This results in the co-elution of the molecules of interest with components of the matrix, which impacts the specificity of the method. Multidimensional (2D) chromatography with heart cutting (which is the transfer of one or more selected groups of compounds eluted from the first gas chromatographic column onto the second) can represent a solution [106] but is usually time consuming. Generally speaking, the use of a high-performance chromatographic method makes it possible to considerably increase the resolution, sensitivity, specificity, and speed of the complete mass spectrometry set-up [106].

Quantification of these steroids requires an optimisation of the quantification method and the use of standards (unlabelled molecules and deuterated molecules or those labelled with deuterium (D), ^13^C, or ^15^N) when they are required. There are two types of quantification: relative and absolute. Relative quantification is often associated with HRMS-based untargeted analyses and consists of comparing the relative abundance of ions along the ordinate axis (intensity of peaks or area of peaks) of a mass spectrum.

This results in an abundance ratio for each molecule between two distinct experimental conditions. This quantification method does not require the use of ranges of labelled and unlabelled standards (as internal or external standards), unlike the absolute quantification method. In MS-based absolute quantification, the amount of the molecule is calculated from the ratio of ion intensities between the analyte and its standard [110]. Absolute quantification methods are much more widely used for the determination of steroids and provide more accurate results.

Tandem mass spectrometer models currently suitable for the quantification of steroids include triple quadrupole (QqQ), tandem quadrupole-time of flight (Q-TOF), and quadrupole-orbitrap (Q-Orbitrap) spectrometers. The ionisation method associated with these tandem spectrometers is electrospray ionisation (ESI) for LC. This ionisation technique, which takes place under atmospheric pressure, mainly induces the formation of monocharged or multicharged ions (z > 1). It can be directly coupled to the output of the LC since the ESI allows the ionization of molecules in solution. ESI is particularly suited to the mild ionisation of low mass molecules, such as steroids [111].

QqQ is the combination of two quadripolar analysers, separated by a collision cell. A quadrupole is a low-resolution analyser, meaning that it allows the selection of ions with a low accuracy in mass (0.1 Da). When two quadrupoles are coupled together and separated by a collision cell (QqQ), the spectrometer can be used in tandem mode. The two quadripoles can be set to “SIM” or “scan” mode. In “SIM” mode, a quadrupole allows only one ion to pass through at an *m*/*z* ratio. In “scan” mode, the quadrupole allows a whole range (also selected in advance) of *m*/*z* ratios to pass through. The combination of these two modes results in four types of procedures.

To quantify, the “multiple reaction monitoring” (MRM) procedure is used: both quadrupoles are in SIM mode. In this set-up, a first precursor ion is selected by the first quadrupole Q1. This is, then, fragmented in the collision cell q2. Finally, a single fragmented ion is selected by the last quadrupole Q3 before reaching the detector. The QqQ, therefore, allows a double selection, ideal for quantification problems [112]. In fact, under these conditions, a highly specific signal is obtained [106], which somewhat overcomes the resolution of a simple LC-ESI-Quadrupole spectrometer. To sum up, QqQ is generally used for targeted quantifications.

Tandem high-resolution mass spectrometers (HRMS), such as Q-TOF (QqTOF) spectrometers and quadrupole-orbitrap (Q-Orbitrap) are composed, respectively, of a quadrupole, a collision cell, and a final “time-of-flight” (TOF) analyser and a quadrupole, a collision cell, and an ion trap based on orbital trapping of the ions in an electrostatic field [113]. The TOF analyser has the particularity of being highly resolving [113]. Moreover, it allows a high mass accuracy (of the order of a few ppm) and a great speed of scanning. Thus, it is possible to collect a large amount of information on many molecules without losing detection sensitivity.

The spectral resolution is also constant over the entire *m*/*z* range, unlike trap or orbitrap type analysers. Orbitrap analysers exhibit higher mass resolution compared with TOF analysers, a good mass accuracy (1–5 ppm), and a high dynamic range [114]. Q-Orbitrap may be applied either in full scan/single ion monitoring or HR-MS acquisition mode [115]. Advantages provided by tandem HRMS make it especially relevant to use for analysis in “full scan” (or “scanning”) mode, although MRM remains possible. Tandem HRMS is, therefore, generally used for non-target profiling of steroids, proving a relative quantification when used in full scan.

However, absolute quantification can also be provided if a range of standards is used. Basing the quantification on the full MS scan in high resolution mode also allows the measurement of the abundance of other molecules with an increased mass accuracy and, thus, to obtain a relative quantification of endogenous compounds at the same time as the quantification of target molecules (e.g., steroids). This is referred to as non-targeted metabolomics by HR-MS.

To evaluate the quality of MS-based quantification of steroids, various characteristics are studied, such as sensitivity, specificity, resolution, measurement accuracy, and dynamic range. These characteristics depend mainly on the (tandem) analysers used. A range of various steroids of known concentrations is injected, and the concentrations calculated by the spectrometer are compared with the actual concentrations [104]. Using the same technique, the linear range is sought. This is the range of concentrations for which the quantification of a steroid remains accurate.

This range may vary from one steroid to another from the same sample [104]. The “ULOL” (“upper limit of linearity”) concentration is the highest concentration for which quantification is correct [106]. Similarly, the LOQ concentration is the lowest concentration for which quantification makes sense. There are other parameters studied, such as the LOD (“limit of detection”), which is especially interesting to determine in the case of qualitative studies [107]. This is the lowest concentration at which a compound can be detected (S/N > 3) [104].

Recently, LC-ESI-QqQ type spectrometry has been the most widely used and most suitable technique for the quantification of steroids due to its high sensitivity, high specificity, and wide dynamic range [106]. However, the recent improvement in the performance of Orbitrap and TOF-type analysers (by increasing their linear range and resolution) has made it possible to match the capabilities of quadrupole analysers. Quantification by LC-ESI-qQTOF has become possible and offers a major advantage: the possibility of analysing a sample in “full scan” mode. Under these conditions, the simultaneous analysis of many steroids, even similar ones, is possible without a loss of sensitivity.

An advantage of LC-ESI-qQTOF instrumentations compared to Orbitrap instruments is the ability to associate ion mobility (IMS-MS) to mass measurements for structure characterisation. Ion mobility in MS is an analytical method used to separate molecules and particularly isomers by measuring the mobility of ions through a collisional gas by using drift tube (DTIMS), travelling wave (TWIMS), trapped (TIMS), field asymmetric waveforms (FAIMS), and cyclic ion mobilities [116].

These different IMS-MS technologies result in different precision in the ion mobility of a molecule that is expressed in collision cross section (CCS) values. The most precise ion mobility is the cyclic ion mobility [117]. CCS measurements are constant values regardless of the mass spectrometer and the ion mobility type used. This allows constituting CCS values databases and introduces a new dimension in small molecule and steroid annotation, in addition to *m*/*z*, chromatographic retention time, MS2 fragments, and isotopologue abundance measurements. IMS allows the separation and characterization of different steroid isomers [103].

All mass spectrometry-based techniques, as described earlier, use different steps of extractions of steroids from tissue and, consequently, are limited when a focus on the spatial distribution of steroids is desired. Mass spectrometry imaging (MSI) is a mass spectrometry technique that allows visualisation of the spatial distribution of molecular species, like lipids, glycans, peptides, and drugs as well as small molecules, like steroids, in tissues or complete organisms [118]. MSI can be used with MALDI (MALDI-IMS), electrospray (DESI), or secondary ion MS (SIMS) ionisations.

The spatial distribution of steroids by MSI was already determined in adrenal gland [119] or mouse testes by using steroid on-tissue derivatisation [120,121]. Increases in spatial resolution, improvement of data processing, quantification methods, and depth profiling represent the challenging areas in MSI [118].

### 6.3. Mass Spectrometry in the Service of Human Fetal Gonad Research

The exploitation of MS for the study of steroidogenesis in the fetal testes began in the 1970s [122], far after the first use of MS for steroid quantification [123]. These studies carried out on gonadal extracts helped to complete the results obtained at the same time, using radiolabeled precursors. GC-MS (and GLC) made it possible to identify and quantify not only T but also its endogenous precursors in gonadal extracts. Indeed, in the pooled testes of foetuses of 10–22 PCW (12–24 GW) in age, nine neutral steroids (unconjugated monosulphates or disulphates) were found, including 3β,16α-dihydroxy-5-androsten-17-one, which is the products of the 16α-hydroxylating activity and previously shown to be present in in vitro studies [122,124].

Thus, it has been possible to demonstrate that the steroidogenesis enzymes present in fetal testicular tissue are active in vivo for the endogenous production of T [122,125]. However, these studies failed to detect and quantify the expected precursors, P4 and 17OH-Preg. The authors hypothesised that a technological hurdle needed to be surmounted and that the limited accumulation of these precursors was undetectable by the MS method of the time. This technological hurdle was overcome much later, due to the use of GC-MS/MS, which has significantly greater sensitivity.

The measurement by LC-MS/MS of T from media from cultivated testes and ovaries confirmed that the absence of detection of T from cultivated human fetal ovaries was not a question of sensitivity [126]. In contrast, the characterisation of steroidogenesis in human fetal testes in the first trimester became clearer and more refined thanks to both better separation of the testes into groups according to their stage of development and advances in sample preparation and MS technology [85]. Indeed, the preparation of samples included grinding in methanol/water, followed by evaporation of the solvent, enzymatic deconjugation of glucuronides and sulfate forms by β-glucuronidase and arylsulphatase, respectively, followed by a double diethyl ether extraction of all total (free + deconjugated) steroids.

The enzymatic deconjugation of glucuronide and sulphate forms before extraction increased the amount of total steroid. This step is necessary because conjugated steroids degrade under the high temperatures required for GC analysis [127]. Androgens and progestogens were separated from estrogens using liquid–liquid partitioning with pentane, followed by quality purification employing silica (SiOH) solid phase extraction cartridges. Finally, the detection and quantification of androgens and estrogens were performed with the combination of a gas chromatograph to a triple quadrupole mass spectrometer [85]. This workflow led to the observation that human fetal testes at 9–10 PCW (11–12 GW) synthesised significantly higher amounts of total steroids than at 6–7 PCW (8–9 GW).

Moreover, fetal testes at 8–9 GW mostly produced P4 and 17OH-P4 whereas fetal testes at 11–12 GW mostly produced T, DHEA, and androstenedione [85]. This study allowed the quantification of additional metabolites, including P4, 7α-hydroxyprogesterone, 17OH-Preg, DHT, E1, and E2, whose concentrations were lower than 1 ug/100 g of testis. Recently, a combination of incubation of samples with deuterated precursor steroids for conversion experiments, and the identification of the resulting steroid metabolites using sensitive LC-MS/MS, helped in the understanding of the steroid biosynthesis pathway in both first trimester testes and ovaries [37].

Imaging mass spectrometry following derivatisation was recently used to visualise T and DHT in adult mouse testes both inside and outside the seminiferous tubules but with a clear accumulation at the surface of Leydig cells [120,121]. In both cases, detection was improved when steroidogenesis was stimulated by treatment with hCG. Interestingly, T was mainly localised within the seminiferous tubules, while DHT was mainly observed in the interstitium/Leydig cells [120]. Undoubtedly, this approach would be relevant in fetal human testis, and, more generally, in fetal testes, due to the possible interplay in steroidogenesis that is possible in mouse fetal testes at least [128].

Measurement of androgens by TurboFlow-LC-MS/MS has only been very recently used to evaluate the impact of drugs intended to alter signalling pathways, such as the Nodal and the Fibroblast Growth Factor pathways in cultivated first trimester testes [82,83]. Inhibition of the Nodal pathway induced a parallel decrease in secreted T and androstenedione and an increase in the secreted precursors 17OH-P4 and P4. This simultaneous analysis of several steroids allowed by mass spectrometry led to the interpretation of treatment-related inhibition of CYP17A1 activity [82].

In the case of FGF9-signaling interference, stimulation of FGF9 signalling resulted in increased levels of 17OH-P4 and P4 without alteration of the T levels in media from testes cultures. This led to the interpretation that 3β-HSD enzyme activity was elevated, despite the apparent lack of increased 3β-HSD protein expression compared to vehicle controls [83]. Interestingly, the opposite strategy, of inhibition of this signalling pathway, had no impact on T and its precursors [83]. Clearly, the fine dissection of several steps of the pathway by mass-spectrometry, associated with in situ detection of proteins, is an important approach in achieving a better understanding of these physiological pathways.

In toxicological studies, the parallel use of T RIA and LC-MS/MS for a whole spectrum of 30 steroids exemplified a strategy of research in which RIA was used to screen the effects on the terminal steroid of the pathway for different concentrations and ages, while sensitive multiplexed mass-spectrometry was used to dissect the steps of alteration of the steroidogenesis pathway [66]. In association with evaluation of enzyme expression levels, This offered the possibility to focus on the fine dissection of the effects only in organs that were sensitive, which is crucial in view of the preciousness of such biological material. Therefore, in this context, RIA and mass-spectrometry were used as complementary methods.

In addition to explant cultures in hanging drops or inserts, the experimental challenging of toxic effects was also addressed by using xenografts experiments [65,66,75,76,78,79,80,82]. In this case, T measurement is possible only if only the male host is castrated, because it is not possible to distinguish steroids from different species. Testosterone measurement by mass spectrometry was used as an endpoint together with host seminal vesicle weight to assess the androgen environment. As for immunological-based methods, it is surprising that the ever-improving sensitivity of MS methodologies is still not exploited to assess a possible alteration in steroid production by human fetal ovaries, although a start has been made to measure estrogen secretion in cultured human fetal ovarian explants [129].

A final observation to make is the need for thinking outside the box in terms of the matrices available when dealing with organs as small as human fetal gonads. One approach that has been used successfully is the measurement of steroids in the waste “flow through” in extraction methods that use columns to sequentially pull DNA and then RNA from samples before yielding protein. While recovery values vary between steroids, the use of subsequent mass-spectroscopy-based steroid data for comparative purposes in particular makes the best use of precious, limited, resources [130,131].

## 7. Beyond the Gonads: A Universe of Possibilities

The human fetal gonads are only part of the picture in terms of normal development in general and reproductive development in particular. For a start, unlike in rodents, in the human foetus, the hypothalamo-pituitary-gonad/adrenal/thyroid axes begin to be operational as early as the late first trimester, with steroid hormones and their metabolites from the gonads, adrenals, liver, and placenta having developmentally important effects elsewhere in the body. The widespread expression of steroidogenic machinery and steroid receptors in the foetus serve to highlight this importance.

Assumptions extrapolated from rodent and adult humans can be misplaced when dealing with the foetus. For instance, techniques similar to those discussed in this review, have been used in parallel to quantify the circulating steroids in fetal cord blood. In the male, it has long been established that a parallelism exists between testicular androgenic capabilities and circulating levels in the cord blood [55,132]. This contrasts to the constant and sexually non-dimorphic circulating DHT levels that were measured [53,133,134].

We now know that this agrees with the findings of Imperato-McGinley [135] concerning the “guevedoce” (penis at 12) mutation. Due to 5-alpha reductase deficiency, these individuals were born apparently female but at puberty became male. Their inability to convert T to DHT was only overcome at puberty when their T levels finally reached a sufficient threshold to induce full masculinisation.

Sex and gender underpin much of what we are as humans, and the more we discover about the processes involved, the more complex they appear to be. Of course, steroid hormones are critical components of the machinery; however, our understanding has now moved far beyond the original concepts [136]. These were that the testis was the “master” endocrine driver of external genital masculinisation in the male, and that the female genitalia can be regarded as a “default” pathway of differentiation.

Subsequent investigations into syndromes around poor or absent establishment of male or female genitalia confirmed the concept that the differentiation of external genitalia requires the local conversion of T into the more potent androgen DHT via steroid 5a-reductase-2 (SRD5A2) activity [137,138], as suggested by Imperato-McGinley [135]. In addition, proper sexual differentiation also depends on the early development of the liver [139], adrenal cortex, and placenta, all contributing to the circulating steroidome. Mutations of several genes of the steroidogenic pathway in the foetus are detrimental to the differentiation of the external genitalia [140,141].

The improvements in understanding of the ability for steroids to be metabolised and/or biotransformed via different pathways has further complicated our understanding of the mechanisms underpinning steroid action. For instance, studies in marsupials identified a steroidogenic pathway for the production of DHT that bypasses T. This is named the “backdoor” or “alternative” pathway in contrast to the “classical” pathways. The bypass of T has made it possible to hypothesise that certain masculinisation anomalies observed in humans, which were not associated with mutations in enzymes of the classical pathway, could be linked to defects in a similar alternative pathway of DHT production [142].

Indeed, focused sequencing of DNA from members of two families with inherited congenital malformations of the external genitalia identified protein function-altering mutations of two aldo-keto reductases, AKR1C2 and AKR1C4 [142]. However, MS/MS analysis revealed that androsterone was the main backdoor androgen found in fetal plasma, compared to un-detectable circulating DHT levels in second trimester male foetuses [131].

In addition, while intermediates of the backdoor pathway were detected in the placenta and the fetal liver, their levels were extremely low inside the testes (unlike T concentrations, which were extremely high), suggesting that this pathway is a minor one in the human fetal testes [131]. The survey of the conversion of several steroid precursors not only in the testes and ovaries but also in the adrenals and genital skin as well as the expression patterns of the relevant enzymes showed that these organs contained efficient machinery to convert androgens via both the classical and the backdoor (alternative) pathways in both sexes [37].

Altogether, these studies indicated that steroidogenesis should not be looked at solely from the gonadal point of view. In fact, the control of the differentiation of the human fetal urogenital tract is not only dependent on gonadal steroids but also on a complex interaction between gonad and non-gonadal tissues. Furthermore, dissection of the steroidogenic machinery and understanding of human developmental deficits enabled us to conclude that the placenta is a key player in the masculinisation of the male foetus.

## 8. Concluding Remarks

Altogether, it is interesting to note that a given technique is not limited to an era, MS was already in use as early as the late 1970s [125], while conversion experiments continue to be used at the time of publication of this review [37]. Each experimental approach addresses specific scientific questions. While conversion experiments can identify the enzymatic steps at play in an organ, MS can now quantify a large spectrum of steroids, the “steroidome”.

Therefore, the choice of a technique should be driven by the specific scientific question/s being posed, and several complementary and/or additive techniques can be used in a single study to generate the data density essential for improved understanding of the complex and interconnected systems in fetal development. Beyond the debates on the sensitivity and reliability of individual approaches [123,143], which concern every assay whatever the technology, the choice of techniques can be guided by their respective strengths and limitations.

For instance, antibody-based methods can be used for screening effects in toxicological studies in many samples, while physiological or altered steroidogenic pathways can be dissected with MS. Currently, MS remains less frequently used compared with immunological methods, possibly because it requires specific machines and technical skills that are not routinely available or accessible in all laboratories. Unfortunately, costs remain a major stumbling block, with basic steroidomic analyses costing hundreds of euros per sample.

The perpetual evolution and refinements of all these methods, even for those that may seem to be the most outdated, are generators of new findings because they have broken down technological barriers. This constant process of serial improvements also poses risks in terms of non-standard protocols and a lack of standardisation, lowering the inter-laboratory reproducibility and, thus, reducing confidence.

Nevertheless, the small number of human fetal ovarian studies with up-to-date techniques could be partly explained by the consequences of a dogma on the testosterone-dominated differentiation of the urogenital tract [136] as well as by the still too-insensitive approaches that are poorly suited to the exceedingly small weights of the human fetal ovaries that can be collected. The main consequence is the relative absence of the investigation of endocrine disruption by xenobiotics in female gonads compared to the male counterpart. In line with this, the gonads should be considered as cogs in the overall steroidogenic endocrine environment, and all target organs should also be investigated.

## Figures and Tables

**Table 1:** Summary of steroid conversion experiments. Pregnenolone (Preg); 17-hydroxy-pregnenolone (17OH-Preg); Progesterone (P4); 17-hydroxy-progesterone (17OH-P4); dehydroepiandrosterone (DHEA); androstenedione; testosterone (T); 5α-dihydrotestosterone (DHT); and estrone (E1).

Year	Testis/Ovary	Endogenous/Produced	Precursor	Steroid Found	Active Enzymes	Reference
1961	Testis 21 Weeks, 24 Weeks	endo	Preg-7α-^3^H	DHEA T	CYP17A1/HSD17B/HSD3B	[23]
1964	Testis (9–11, 12–15 and 19 Weeks)	endo	4-^14^C-P4	T 17α-hydroxyprogesterone 16α-hydroxyprogesterone	CYP17A1 HSD17B	[26]
	Ovaries (9–11, 12–15 and 19 Weeks)	endo	4-^14^C-P4	20α-hydroxy-4-pregnene- 3-one (only 19 Weeks ovary)		
1965	Ovaries (11 Weeks)	Culture 4–8 days	7-^3^H-P4	7-^3^H-20α-hydroxy-pregnene-3-one		[27]
1966	Testis (21 cm)	Culture 8 days	1-^14^C-sodium acetate	C21: 3β-hydroxy-pregnene-20-one 17-hydroxy-pregnene-3, 20-dione P4 C19: androstene-3, 17-dione T		[28]
1972	Testis mid-gestation	endo	^14^C-sodium acetate	Preg, Preg-sulphate DHEA, DHEA-sulphate D5-androstenediol, T		[29]
1974	Testis (16–20 Weeks)	Endo (foetus perfusion)	4-^14^C-P4	T, androstenedione		[30]
	Ovaries (16–20 Weeks)	Endo (foetus perfusion)	4-^14^C-P4	Neither testosterone nor androstenedione		
1974	Testes		7α-^3^H-Preg	T		[24]
	(1–21 cm)		1,2-^3^H-P4	T		
	Ovaries (1–21 cm)		7α-^3^H-Preg 1,2-^3^H-P4	No testosterone		
1975	Testes	endo	^3^H-Preg-sulphate	DHEA, T, androstenedione		[31]
1975	Ovaries (14–42 Weeks)		4-^14^C-Preg	P4, 17OH-Preg DHEA		[32]
1978	Testes (1–20 cm)	endo	radiolabeled androgen	No estrogen		
	Ovaries (1–20 cm)	endo	l,2,6,7-^3^H-T	E1 and E2 (by the 3.1–5-cm stage)	CYP19A1	[25]
			l,2,6,7-^3^H-androstenedione	E1 and E2 (by the 3.1–5-cm stage)	CYP19A1	
1982	Ovaries	endo	^14^C-Preg	P4, 17OH-Preg, 5-pregnene-3β,20α-diol 5-pregnene-3β,17α,20α-triol 5α-pregnan-3,20-dione	5-ene-3β-HSD 17-hydroxylase 20α-HSD 5α-reductase	[33]
1982	Testis (32-weeks)	endo	^3^H-Preg	androst-5-ene-3α,17β-diol 3β-hydroxyandrost-5-ene-17-one	no activity of 5 α-reductase	[34]
			^3^H-P4	Androstenedione 17b-hydroxy-5a-androstan-3-one Unidentified steroid	no activity of 5 α-reductase	
1984	Testis	endo	^3^H-androstenedione	T	HSD17B	
			^3^H-P4	17OH-P4	HSD17B	[35]
2003	Testis microsomes	endo	17OH-Preg	DHEA	CYP17A1 Δ5 preferred pathway (11-fold)	[36]
			17OH-P4	androstenedione	CYP17A Δ4 pathway	
2019	Testis explants (6–10 WPC)		Deuterated 17OH-P4	Androstenedione T 5α-17OH-pregnanolone	CYP17A1 AKR1C1/3 SRD5A1	[37]
			Deuterated 5α-17OH-pregnanolone	5α-androsterone 5α-andro-stanediol 5α-andro-standione	CYP17A1 AKR1C/3 HSD17B6	
			Deuterated 5α-androsterone	5α-andro-standione 5α-andro-stanediol	HSD17B6 AKR1C1/3	
			Deuterated 5α-androstanediol	5α-androsterone 5α-andro-standione DHT	HSD17B6	
	Ovaries explants (6–10 WPC)		Deuterated 17OH-P4	Androstenedione 5α-17OH-pregnanolone	CYP17A1 AKR1C1/3 SRD5A1	
			Deuterated 5α-17OH-pregnanolone	5α-androsterone 5α-andro-stanediol	CYP17A1 AKR1C/3	
			Deuterated 5α-androsterone	5α-andro-standione DHT 5α-andro-stanediol	HSD17B6 AKR1C1/3	
			Deuterated 5α-androstanediol	5α-androsterone DHT	HSD17B6	

## Supplementary Materials

The following are available online at https://www.mdpi.com/article/10.3390/ijms22136681/s1.
