# Supplementary material for: Six Decades of Research on Human Fetal Gonadal Steroids
Source: Int J Mol Sci. 2021 Jun 22;22(13):6681. doi: 10.3390/ijms22136681 (PMC8268622; doi:10.3390/ijms22136681)
Supplement: Supplementary file 1 [file ijms-22-06681-s001.zip › ijms-1200730-supplementary.pdf]

**Supplemental Table S1:** Guide to nomenclature used for steroids included in this review, including abbreviation, full names and commonly used synonyms.

| Abbreviation | Steroid name | Full name | Common synonyms |
|---|---|---|---|
| Preg | pregnenolone | pregn-5-en-3β-ol-20-one | 3beta-Hydroxypregn-5-en-20-one |
| 17OH-Preg | 17 hydroxy-pregnenolone | 17α-hydroxypregnenolone | 17-Hydroxypregnenolone<br>17-OH-pregnenolone<br>17-alpha-Hydroxypregnenolone |
| P4 | progesterone | pregn-4-ene-3,20-dione | Agolutin<br>Luteohormone |
| 17OH-P4 | hydroxy-progesterone | 17α-hydroxyprogesterone | 17-Hydroxyprogesterone<br>17a-Hydroxyprogesterone |
| DHEA | dehydroepiandrosterone | 3β-hydroxy-androst-5-ene-17-one | Dehydroisoandrosterone |
| | androstenedione | 4-androstene-3,17-dione | Androst-4-ene-3,17-dione<br>4-Androstenedione |
| | androstenediol | 3β,5α-androstenediol | 5α-androstenediol<br>androst-5-ene-3β,17β-diol |
| | androstenedione | androst-4-ene-3,17-dione | 4-androstenedione; Δ4-dione<br>Androst-4-ene-3,17-dione;<br>4-Androstene-3,17-dione<br>17-Ketotestosterone<br>17-Oxotestosterone |
| T | testosterone | 17β-hydroxyandrost-4-en-3-one | 17-beta-Hydroxy-delta(sup 4)-androsten-3-one<br>17-beta-Hydroxyandrost-4-en-3-one<br>17-Hydroxy-(17-beta)-androst-4-en-3-one |
| DHT | dihydrotestosterone | 5α-androstan-17β-ol-3-one | 5α-dihydrotestosterone, 5α-DHT<br>Androstanolone<br>Stanolone, Andractim<br>Androlone |
| | 5α-dihydroprogesterone | 5α-pregnane-3,20-dione | 5alpha-Pregnane-3,20-dione<br>3,20-Allopregnanedione<br>5a-Pregnane-3,20-dione<br>5α-Pregnanedione<br>5-alpha-Dihydroprogesterone |
| 17-OH-DHP | 17α OH dihydroprogesterone | 5α-pregnane 17α-ol-3,20-dione | (5alpha)-17-Hydroxypregnane-3,20-dione<br>Allopregnane-3,20-dione-17α-ol<br>5alpha-Pregnan-17-ol-3,20-dione<br>17α-Hydroxy-5α-pregnane-3,20-dione<br>17-Hydroxydihydroprogesterone<br>17α-hydroxy-dihydroprogesterone |
| | 5α-androstanedione | 5α-androstane-3,17-dione | Dihydroandrostenedione<br>Dihydroandrostendione<br>Androstanedione<br>5alpha-Androstan-3,17-dione |
| | allopregnanolone | 5α-pregnane 3α-ol,20-one | Brexanolone<br>Allopregnan-3alpha-ol-20-one<br>Allotetrahydroprogesterone |
| 17-OH-allo | 17-OH allopregnanolone | 5α-pregnane 3α,17α-diol, 20-one | (3α,5α)-3,17-Dihydroxypregnan-20-one<br>5α-pregnan-3α,17α-diol-20-one<br>3α,17-dihydroxy-5α-pregnan-20-one |

| | | | |
|---|---|---|---|
| | | | 3$\alpha$,5$\alpha$-3,17-dihydroxypregnan-20-one<br>17-hydroxyallopregnanolone. |
| | androsterone | 3$\alpha$-hydroxy-5$\alpha$-androstan-17-one | 5$\alpha$-androstan-3$\alpha$-ol-17-one<br>Androkinine<br>Androtine |
| Adiol (3b-diol) | Androstanediol (3b-diol) | 5$\alpha$-androstane-3$\beta$,17$\beta$-diol | 3$\beta$-Androstanediol<br>Androstane-3,17-diol<br>(3$\beta$,5$\alpha$,17$\beta$)-Androstane-3,17-diol<br>3$\beta$-Diol; Maxterone<br>3$\beta$,17$\beta$-Androstanediol<br>3$\beta$,17$\beta$-Dihydroxy-5$\alpha$-androstane |
| Adiol (3a-diol) | Androstanediol (3a-diol) | 5$\alpha$-androstane-3$\alpha$,17$\beta$-diol | 3$\alpha$-Androstanediol<br>Androstane-3,17-diol, (3$\alpha$,5$\alpha$,17$\beta$)-<br>(3$\alpha$,5$\alpha$,17$\beta$)-Androstane-3,17-diol<br>3$\alpha$,17$\beta$-Dihydroxy-5$\alpha$-androstane<br>Androstane-3$\alpha$,17$\beta$-diol |
| E2 | Estradiol | estra-1,3,5(10)-triene-3,17$\beta$-diol | Oestradiol;<br>17$\beta$-Estradiol; 17$\beta$-Oestradiol |
| E1 | Estrone | 3-hydroxyestra-1,3,5(10)-trien-17-one | Oestrone;<br>folliculin |
| E3 | Estriol | estra-1,3,5(10)-triene-3,16$\alpha$,17$\beta$-triol | Oestriol; Estratriol;<br>Trihydroxyestrin;<br>Trihydroxyoestrin;<br>16$\alpha$-Hydroxyestradiol |

**Supplemental table 2:** Guide to steroidogenesis nomenclature used for steroidogenic machinery elements included in this review, including alternative and commonly used names. The reader should beware the frequent differences between gene and protein names and the range of alternative names. In the text of this review we have used the most commonly used names.

| **Gene symbol** | **Gene name** | **Protein name** | **Alternative names** |
|---|---|---|---|
| *AKR1C1* | aldo-keto reductase family 1 member C1 | Aldo-keto reductase family 1 member C1 | 20 alpha-hydroxysteroid dehydrogenase (20-alpha-HSD); aldo-keto reductase C ; |
| *AKR1C2* | aldo-keto reductase family 1 member C2 | aldo-keto reductase family 1 member C2 | 3-alpha hydroxysteroid dehydrogenase, type III (3-alpha-HSD3) |
| *AKR1C3* | aldo-keto reductase family 1 member C3 | aldo-keto reductase family 1 member C3 | 3-alpha hydroxysteroid dehydrogenase, type II; testosterone 17-beta-dehydrogenase 5 (HSD17B5); prostaglandin F synthase (PGFS) |
| *CYB5A* | cytochrome b5 type A | cytochrome b5 | CYB5; MCB5; METAG |
| *CYP7B1* | cytochrome P450 family 7 subfamily B member 1 | cytochrome P450 7B1 | 24-hydroxycholesterol 7-alpha-hydroxylase; 3-hydroxysteroid 7-alpha hydroxylase |
| *CYP11A1* | cytochrome P450 family 11 subfamily A member 1 | cholesterol side-chain cleavage enzyme, mitochondrial | cytochrome P450 11A1 (CYP11A); cytochrome P450(scc) (P450SCC) |
| *CYP17A1* | cytochrome P450 family 17 subfamily A member 1 | steroid 17-alpha-hydroxylase/17,20 lyase | 17-alpha-hydroxyprogesterone aldolase; cytochrome P450 17A1 (P450C17); cytochrome P450-C17 (CYP17) |
| *CYP19A1* | cytochrome P450 family 19 subfamily A member 1 | aromatase | Cytochrome P-450AROM (ARO); Cytochrome P450 19A1 (CYP19A1, CYP19; P-450AROM); Estrogen synthase |
| *HSD3B1* | hydroxy-delta-5-steroid dehydrogenase, 3 beta- | 3 beta-hydroxysteroid dehydrogenase/Delta 5-->4-isomerase type 1 | HSD3B; 3BetaHSD |

| | | | |
|---|---|---|---|
| | and steroid delta-isomerase 1 | | |
| *HSD3B2* | hydroxy-delta-5-steroid dehydrogenase, 3 beta- and steroid delta-isomerase 2 | 3 beta-hydroxysteroid dehydrogenase/Delta 5-->4-isomerase type 2 | HSDB; HSD3B |
| *HSD17B2* | hydroxysteroid 17-beta dehydrogenase 2 | 17-beta-hydroxysteroid dehydrogenase type 2 | 17-beta-HSD 2; HSD17;<br>20 alpha-hydroxysteroid dehydrogenase (20-alpha-HSD);<br>estradiol 17-beta-dehydrogenase 2 (EDH17B2);<br>testosterone 17-beta-dehydrogenase;<br>short chain dehydrogenase/reductase family 9C member 2 (SRD9C2) |
| *HSD17B3* | hydroxysteroid 17-beta dehydrogenase 3 | testosterone 17-beta-dehydrogenase 3 | 17-beta-hydroxysteroid dehydrogenase type 3;<br>17-beta-HSD 3;<br>testicular 17-beta-hydroxysteroid dehydrogenase<br>estradiol 17-beta-dehydrogenase 2 (EDH17B3);<br>short chain dehydrogenase/reductase family 12C member 2 (SDR12C2); |
| *HSD17B6* | hydroxysteroid 17-beta dehydrogenase 6 | 17-beta-hydroxysteroid dehydrogenase type 6 | 17-beta-HSD 6;<br>3(alpha->beta)-hydroxysteroid epimerase (HSE);<br>short chain dehydrogenase/reductase family 9C member 6 (SDR9C6) |
| *HSD17B10* | hydroxysteroid 17-beta dehydrogenase 10 | 3-hydroxyacyl-CoA dehydrogenase type-2 | 17b-HSD10;<br>3-hydroxy-2-methylbutyryl-CoA dehydrogenase;<br>amyloid-beta peptide binding alcohol dehydrogenase ; AB-binding alcohol dehydrogenase (ABAD)<br>endoplasmic reticulum-associated amyloid beta-peptide-binding protein (ERAB)<br>mitochondrial RNase P subunit 2<br>mitochondrial ribonuclease P protein 2<br>short chain L-3-hydroxyacyl-CoA dehydrogenase type 2 (SCHAD) |
| RDH16 | retinol dehydrogenase 16 | retinol dehydrogenase 16 | human epidermal retinol dehydrogenase (hRDH-E); sterol/retinol dehydrogenase;<br>microsomal NAD+-dependent retinol dehydrogenase 4 (RODH-4)<br>short chain dehydrogenase/reductase family 9C, member 8 (SDR9C8) |
| RDH5 | retinol dehydrogenase 5 | retinol dehydrogenase 5 | HSD17B9<br>11-cis RDH ; 11-cis RoDH ;<br>9-cis retinol dehydrogenase (9cRDH);<br>retinol dehydrogenase 1 (RDH1)<br>short chain dehydrogenase/reductase family 9C member 5 (SDR9C5) |
| *SRD5A1* | steroid 5 alpha-reductase 1 | 3-oxo-5-alpha-steroid 4-dehydrogenase 1 | steroid 5-alpha-reductase type I (S5AR1);<br>steroid-5-alpha-reductase, alpha polypeptide 1 (3-oxo-5 alpha-steroid delta 4-dehydrogenase alpha 1) |
| *SRD5A2* | steroid 5 alpha-reductase 2 | 3-oxo-5-alpha-steroid 4-dehydrogenase 2 | type II 5-alpha reductase; 5 alpha-SR2; S5AR 2;<br>SR type 2 |

| | | | |
|---|---|---|---|
| | | | steroid-5-alpha-reductase, alpha polypeptide 2 (3-oxo-5 alpha-steroid delta 4-dehydrogenase alpha 2) |
| *SRD5A3* | steroid 5 alpha-reductase 3 | polyprenol reductase | 3-oxo-5-alpha-steroid 4-dehydrogenase (NADP(+));<br>3-oxo-5-alpha-steroid 4-dehydrogenase 3<br>S5AR 3; SR type 3 |
| *STAR* | steroidogenic acute regulatory protein | steroidogenic acute regulatory protein, mitochondrial | START domain containing 1 (STARD1); START domain-containing protein 1<br>StAR related lipid transfer (START) domain containing 1<br>cholesterol trafficker<br>mitochondrial steroid acute regulatory protein<br>steroidogenic acute regulator<br>testis secretory sperm-binding protein Li 241mP |
| *STS* | steroid sulfatase | steryl-sulfatase | arylsulfatase C (ARSC; ASC; ARSC1);<br>estrone sulfatase (ES);<br>steroid sulfatase (microsomal), isozyme S (SSDD)<br>steryl-sulfate sulfohydrolase |
| *SULT2A1* | sulfotransferase family 2A member 1 | sulfotransferase 2A1 | alcohol/hydroxysteroid sulfotransferase (HST, STD)<br>bile-salt sulfotransferase 2A1 (ST2A1; ST2A3)<br>sulfotransferase family, cytosolic, 2A (ST2; hSTa; SULT2A3)<br>dehydroepiandrosterone (DHEA)-preferring, member 1 (DHEA-ST; DHEA-ST8, DHEAS) |
| *SULT2B1* | sulfotransferase family 2B member 1 | sulfotransferase 2B1 | ST2B1<br>alcohol sulfotransferase<br>hydroxysteroid sulfotransferase 2 (HSST2)<br>hydroxysteroid sulfotransferase SULT2B1a<br>sulfotransferase family, cytosolic, 2B, member 1 |
| *UGT2B7* | UDP glucuronosyltransferase family 2 member B7 | UDP-glucuronosyltransferase 2B7 | 3,4-catechol estrogen-specific UDPGT<br>UDP glucuronosyltransferase 2 family, polypeptide B7 (UDPGT2B7)<br>UDP-glucuronosyltransferase 2B9 (UDPGT 2B9; UGT2B9)<br>UDP-glucuronyltransferase, family 2, beta-7 (UDPGT 2B7; UDPGTH2; UDPGTh-2) |
| *UGT2B15* | UDP glucuronosyltransferase family 2 member B15 | UDP-glucuronosyltransferase 2B15 | UDP glucuronosyltransferase 2 family, member 15; UDP glycosyltransferase 2B15; UDP glucuronosyltransferase 2 family, polypeptide B15; UDP gycosyltransferase 2 family, member B15 ; UDP-glucuronosyltransferase UGT2B15; UDP-glucuronyltransferase, family 2, beta-15 (UDPGT2B15)<br>UDP glucuronosyltransferase 2 family, member B8; UDP-glucuronosyltransferase 2B8<br>uridine diphosphate glucuronosyltransferase 2 family, member B8 (UGT2B8; UDPGT 2B8)<br>uridine diphosphate glycosyltransferase 2 family, member B15 |
| *UGT2B17* | UDP glucuronosyltransferase family 2 member B17 | UDP-glucuronosyltransferase 2B17 | C19-steroid-specific UDP-glucuronosyltransferase |

| | |
|---|---|
| | UDP glucuronosyltransferase 2 family, polypeptide B17 (UDPGT2B17)<br>UDP glycosyltransferase 2 family, member B17<br>UDP-glucuronyltransferase, family 2, beta-17 |
